# Investigating Cardiovascular Risk as a Moderator of the Relationship between Blood‐Based Alzheimer's Disease Biomarkers and Cognitive Impairment – A Cross‐sectional Analysis of Data from the Bio‐Hermes Study

**DOI:** 10.1002/alz70856_096389

**Published:** 2025-12-24

**Authors:** Angelina Kirilova Kancheva

**Affiliations:** ^1^ University of Glasgow, Glasgow, Glasgow, United Kingdom

## Abstract

**Background:**

Blood‐based biomarkers show great promise to revolutionize diagnosis and prognosis of Alzheimer's disease (AD) and other dementias. Cardiovascular disease (CVD) risk factors, such as hypertension and diabetes, contribute to risk of cognitive impairment (CI), vascular and Alzheimer's disease dementia. The relationship between CVD risk and blood‐based biomarkers for AD remains unclear. We sought to explore whether CVD risk significantly moderates the relationship between blood‐based AD biomarkers and CI.

**Method:**

We included participants with mild cognitive impairment or probable AD (CI group), versus cognitively normal comparators (CN) from the Bio‐Hermes study. Clinical presentation of CI was determined based on clinical screening procedures. CVD risk was calculated using the Atherosclerotic CVD (ASCVD) risk score calculator. We conducted a series of logistic regression analyses to evaluate the association of each of several AD biomarkers (plasma amyloid beta (Aβ) 42/Aβ40, phosphorylated tau (*p*‐tau)181, *p*‐tau217, apolipoprotein E ε4 allele (Apoε4) status) and CVD risk, with CI. We then tested moderation by CVD risk in each model.

**Result:**

We included 661 participants (*n* = 266 CN, n = 395 CI; mean age 72.4 years; 55% male). In each of the models, blood‐based biomarkers and CVD risk were significantly associated with CI. Strongest association was for *p*‐tau217 (OR = 2.34 [95% CI:1.89‐2.9]). CVD risk moderated the relationship between *p*‐tau181 and CI (OR = 0.78 [95% CI: 0.64‐0.95]) but no moderation effect was observed for any of the other biomarkers.

**Conclusion:**

In our study, blood‐based dementia biomarkers and CVD risk significantly associated with CI, but CVD risk did not moderate the relationship between blood‐based biomarkers and CI. Blood‐based biomarkers and CVD risk might confer independent risk of CI. Given the multiple risk factors for dementia and large implications for social and healthcare systems globally, CVD risk assessment should complement other dementia biomarker assessments for accurate and expeditious prognosis.

